# T Cells and Cancer: How Metabolism Shapes Immunity

**DOI:** 10.3389/fimmu.2016.00020

**Published:** 2016-02-01

**Authors:** Barbara Molon, Bianca Calì, Antonella Viola

**Affiliations:** ^1^Department of Biomedical Sciences, University of Padua, Padua, Italy; ^2^Venetian Institute of Molecular Medicine (VIMM), Padova, Italy

**Keywords:** tumor microenvironment, T lymphocyte activation, immune escape, reactive nitrogen species, immunotherapy

## Abstract

Tumor microenvironment is characterized by a consistent reduction in oxygen and blood-borne nutrients that significantly affects the metabolism of distinct cell subsets. Immune cells populating malignant lesions need to activate alternative pathways to overcome tumor-prolonged nutrient deprivation. In particular, the metabolic switch occurring in transforming tissues dramatically impacts on tumor-infiltrating T cell biology. Remarkably, the recruitment and activation of T cell within cancers are instrumental for effective antitumor response. Therefore, T cell metabolic adaptation acts as crucial checkpoint hijacked by tumors to dampen antitumor immunity.

## Introduction

Cancers are not mere collections of relatively homogenous tumor cells, but they rather form a sort of crowded organ composed of different cell populations supporting malignant nourishment and progression. To ensure tumor growth and immune evasion, the stromal component of tumor mass undergoes numerous metabolic adaptations, reprograming the mode of energy generation (1). Metabolic reprograming of cancer and stromal cells fulfils the urgent need for energy supply to support tumor cell proliferation and progression, thus representing one additional hallmark of cancers (2). Notwithstanding, the first observations of metabolic alterations in cancer cells date back to the early 90s, when Otto Warburg found that cancer cells, regardless of oxygen tension, prefer to metabolize glucose by glycolysis.

Albeit more rapid than oxidative phosphorylation, anaerobic glycolysis (fermentation) is less efficient in ATP generation and, reducing lactate, contributes to the accumulation of metabolites that promote immunosuppression (3). The glycolytic switch of tumor cells, also known as “Warburg effect” offers a valuable tool for diagnosis, staging, and monitoring therapy response in many cancers, and it accounts for the physiological basis for positron emission tomography (PET) in clinical oncology (4). In addition to the Warburg effect, both tumor and stromal cells exploit other catabolic routes aimed at amino acid conversion into more affordable energetic products, as well as change in lipid metabolism (5).

This metabolic adaptation works indeed as crucial checkpoint hijacked by tumors to dampen antitumor immunity. Solid tumors build up a forbidding environment characterized by a consistent reduction in O_2_ and blood-borne nutrients. The paucity of appropriate nutrients represents, so far, a limiting step for the effectiveness of antitumor immune responses since T cells infiltrating malignant tissues need to face the tumor hostile environment to exert their functions.

## Metabolic Adaptations of T Cells within the Tumor

Tumor-infiltrating immune cells include cell subsets belonging to both the innate and the adaptive arms of the immune system. The immune setting within the locoregional tumor microenvironment significantly dictates cancer fate. A strong lymphocytic infiltration, so far, has been reported to be associated with good clinical outcome in different human tumors (6). The intratumoral activation of T cell responses may result in the control of tumor growth and spreading in some cancers, such as in melanoma (7). In fact, recent advances in cancer immunotherapy based on the clinical exploitation of monoclonal antibodies targeting T cell-immune checkpoints, such as PD-1 (8) and CTLA4 (9), clearly confirmed the requirement of an efficient T cell activation for an effective antitumor response. CD4^+^ and CD8^+^ T lymphocytes are fundamentally able to recognize tumor antigens and to activate their effector programs even within transformed tissues.

T cells, experiencing the metabolic framework of growing tumors, need to activate distinct pathways to accomplish their functional requirements. Nonetheless, tumors impose several limitations to dampen T cell immunity, and among these, the control of nutrient availability and handling represents one crucial process. In growing tumors, where neoplastic cells proliferate at a very high rate, the deprivation of particular nutrients, such as glucose or amino acids, from the environment dramatically hinders T cell functions. Basically, T cell metabolic reprograming relies upon the activation of distinct transcriptional and signaling pathways that are now beginning to be elucidated.

Metabolic reprograming accounting for the increasing energy demand is crucial for the triggering of T cell effector functions. The transition from the “energy-saving” oxidative metabolism – typical of naïve/memory T cells – to the primarily biosynthetic and anabolic metabolism of effector T cells requires a substantial uptake of nutrients from the environment (10, 11). This is clearly connected to the urgency of an activated T cell to support its own duties, such as cell proliferation, cytotoxicity, and adaptive cytokine production. To do that, with respect to quiescent cells, effector T lymphocytes switch the metabolism toward aerobic glycolysis increasing the uptake of glucose and glutamine from the outside and the consumption of oxygen (12). In particular, the engagement/disengagement of aerobic glycolysis represents a crucial mechanism controlling T cell effector status by means of the posttranscriptional regulation of IFN-γ production (13). Furthermore, the activation of PD-1 signal, which is linked to T cell exhaustion, inhibits the uptake and utilization of glucose and glutamine increasing the rate of fatty acid β-oxidation (FAO) in T cells (14). A drop of glucose level within tissues leads T cells to enter a dormant “anergy” state to spare energy (15) or to preferentially activate autophagy as survival mechanism (16) to counteract nutrient insufficiency. Activated T cells upregulate the surface expression of key nutrients receptors, such as the amino acid and glucose transporters, triggering the hypoxia-inducible factor-α (HIF1-α), c-Myc, and the PI3K/Akt/mTOR (the mammalian target of rapamycin) (17, 18), which play a pivotal role in energetic adaptations of both cancer and immune cells (19).

HIF1-α is a transcriptional factor that is upregulated when oxygen tension decreases. In normoxic conditions, HIF1-α is constantly degraded by the proteasome complex; conversely, during hypoxia, it is stabilized and regulates the expression of angiogenic and tissue remodeling factors (20), together with enzymes involved in the commitment to glycolysis. Upon activation, HIF1-α promotes the expression of lactate dehydrogenase, pyruvate dehydrogenase kinase PDK1 (21), and several glycolysis-related genes, such as GLUT1 and PFKFB3 (22), with the consequence of increasing glucose uptake and glycolysis, reducing pyruvate flux into the TCA cycle, oxidative phosphorylation, and oxygen consumption (20). Interestingly, it has been recently reported that HIF-1α represents a crucial metabolic checkpoint for the differentiation of either T_reg_ or Th17 cells (23). Commonly associated with T_regs_, the CD4 Th17 cell subset has increasingly gained attention in cancer immunity.

In combination with HIF1-α, the oncogene c-Myc also activates the expression of glucose transporters, PDK1 and lactate dehydrogenase A (LDHA), which is responsible for enhancing glucose influx and glycolysis. Moreover, c-Myc induces the expression of glutamine transporters and glutaminase1 for glutaminolysis (24). It has been reported that activated T cells exploit a non-canonical Myc-dependent pathway coupling glutaminolysis to polyamine biosynthesis in order to sustain cell growth and proliferation in both pathological and physiological situations (25).

mTOR is a downstream target of the PI3K–AKT signaling and has been demonstrated to enhance the expression of HIF1-α in immune cells recruited at the tumor lesion (26).

In tumours, mTOR activation promotes glycolysis by enhancing (HIF1) activity; additionally it sustains fatty acid and protein synthesis thus supporting the survival and functions of both malignant and pro-tumoural immune cells of (27, 28). Within solid tumors, mTOR activation tunes the balance between effector versus memory CD8 T cells by regulating the expression of the transcription factor T-bet (29).

An increased AMP:ATP ratio induces the activation of the energy sensor AMPK, which plays a key, but still controversial, role in T cell antitumor immunity. AMPK activation in T cells may resemble its role in tumor cells, where it controls cell viability and proliferation under poor nutrient conditions (30). Additionally, T cells may exploit this nutrient sensor to rapidly face glucose limitation in the environment. In this regard, it has been recently reported that *in vivo* the AMPK signaling pathway regulates *Ifng* mRNA translation and the glutamine-dependent mitochondrial metabolism in T cells (31). Moreover, recent findings showed that the selective deletion of AMPK in T cells decreases IFNγ and Granzyme B production in intratumoral CD8^+^ T cells (32).

In main contrast to effector T cells that metabolically suffer the tumor nutrient landscape, other T cell subsets, such as T regulatory cells (T_regs_), feel comfortable with the very same environment. This is probably due to the abundance of growth factors (such as transforming growth factor-β) (33) and chemokines (such as CCL22) (34) promoting T_reg_ differentiation and recruitment. The presence of T_regs_ in solid tumors essentially correlates with poor prognosis (35). In particular, in ovarian cancer, a higher CD8^+^ T cells/T_reg_ cells ratio associates with a particularly favorable clinical outcome (34). Nonetheless, T_reg_ contribution in the context of chronically inflamed tissues, such as in colorectal cancer (CRC), remains controversial. Discordant evidence in patients with CRC support, so far, the notion that T_reg_ infiltration and accumulation in cancerous tissues may play either a negative (36, 37) or positive (38, 39) predictive role for patient survival. Metabolically, T_regs_ do not require high rate of glucose consumption and usually express low level of the Glut1 transporter (40). Natural and Inducible T_regs_ are primarily oxidative and metabolize pyruvate through the TCA cycle. They preferentially utilize lipid beta-oxidation and present high levels of activated AMPK, which is usually active in starved-fed conditions (41, 42). Furthermore, it has been reported that several intratumoral metabolic leftover as lactate and kynurenine support T_reg_ differentiation while suppressing T cell cytotoxic activity (43). The generation of T_reg_ cells *in vivo* is dependent on the aryl hydrocarbon receptor (AHR)-mediated induction of IDO1 and kynurenine. AHR is ligand-activated transcription factor, which is chronically activated in aggressive tumors. Therefore, in contrast to T effector cells, T_regs_ feel comfortable in the nutrient-restrictive tumor microenvironment, where they can efficiently active immunosuppressive pathways. Additionally, tumor-derived lactate polarizes immune responses toward a Th17/Th23 phenotype (3, 44).

## Tumor Metabolism Drives T Cell Dysfunction

Tumor progression is characterized by a tangled network of relationships among different cell types that collectively exploit a metabolic reprograming and mutually influence their functionality and, in particular, T cell functions. Because of the Warburg effect and high glucose consumption by cancer cells, tumor microenvironment shows reduced extracellular concentration of glucose (45). As a consequence of glucose deprivation, tumor-infiltrating T cells decrease aerobic glycolysis and generation of the phosphoenolpyruvate (PEP) metabolite involved in TCR-dependent activation of Ca^2+^ and NFAT signaling, thus losing their antitumoral effector functions (46). Moreover, lactate accumulation in the microenvironment has been shown to affect T cell activation by impairing lactic acid secretion and disturbing metabolism. In detail, tumor acidosis is accompanied by the suppression of proliferation and cytokine production in cytotoxic T cells (CTLs) and finally inhibits CTL cytotoxic activity (47). Acidification of tumor microenvironment dramatically impairs cytotoxic T cell proliferation and function (48), though it does not affect T_regs_ (41), inhibits monocyte-derived DC differentiation and activation, and is positively correlated with radioresistance (49). Accelerated fermentation, generating high level of lactate, constitutes indeed a marker for metastases and correlates with poor prognosis (50). Also hypoxia represents a hindrance to T cell antitumor responses. HIF-1α has been shown to upregulate the expression of PD-1 ligand on cancer cells, thus inhibiting T cell-mediated cytotoxicity (51). Beyond glycolysis, amino acid metabolism represents a crucial process in tumor progression. In particular, l-arginine and tryptophan catabolism have been demonstrated to be dysregulated in cancers (5, 52).

l-Arginine metabolism is strictly dependent on the activity of the enzymes, nitric oxide synthase (NOS) and arginase (ARG). While NOS oxidizes arginine to citrulline and nitric oxide (NO), arginase hydrolyzes arginine into ornithine and urea. Several reports have showed the expression of the inducible isoform of NOS enzyme (iNOS) in human colon cancers, ovarian and prostate cancers, melanoma, and other malignant lesions, including the hematological ones (53). Similarly, ARG activity is upregulated in colon, breast, lung, and prostate cancer (54), and ARG1 is associated with M2 polarized, protumoral TAMs (55). The activation of both enzymes generates high levels of NO capable of either promoting or inhibiting tumor progression or metastasis, depending on the local concentration and duration of exposure, cellular sensitivity and hypoxia/re-oxygenation process within tumor microenvironment (48). Additionally, NO produced by iNOS may rapidly react with reactive oxygen species within the tumor lesion and produce reactive nitrogen species (RNS) such as peroxynitrite, which is very toxic for many cells, and in particular for T cells. Peroxynitrite induces apoptotic cell death in lymphocytes by interfering with protein tyrosine phosphorylation *via* nitration of tyrosine residues (56) or by nitrating the voltage-dependent anion channel, a component of the mitochondrial permeability transition pore (57). Although solid tumors are characterized by lymphocyte infiltration, tumor-infiltrating lymphocytes frequently are unable to kill autologous tumor cells, experiencing an anergic/tolerant state (58, 59). Importantly, we have previously shown the presence of high levels of nitrotyrosines in both cancer specimens and tumor-infiltrating lymphocytes (60). As a consequence of high rate of RNS production, the tumor microenvironment is not suitable for T cell functions, and indeed a number of reports indicate that peroxynitrite negatively affects T-cell-mediated immunity within the tumor (61) and that tumor-infiltrating lymphocytes have defects in both signal transduction and effector killing capacity (62).

Unlike normally responsive lymphocytes in healthy tissue and peripheral blood, tumor-infiltrating lymphocytes are not activated locally by powerful signals acting either on TCR or downstream signaling pathways. We have previously reported that this dormant state is dependent on the enhanced intratumoral metabolism of l-arginine, because the simple addition of arginase and NOS-specific inhibitors was sufficient to rouse CTLs, activate them, and start a number of events leading to cytolytic granule polarization and killing of cognate targets (60). Furthermore, high and prolonged exposure to RNS has been demonstrated to modulate tyrosine phosphorylation of several proteins, such as the CD3ζ chain of the TCR complex, and release of Ca^2+^ from intracellular stores, thus promoting downregulation of membrane receptors, such as CD4, CD8, and chemokine receptors from T cells (63). Moreover, RNS dampen antitumor immunity by generating post-translational modifications – nitration and nitrosylation – of key proteins for T cell activation (61) and recruitment to the tumor site (64). Post-translational modifications of chemokines and chemokine receptors constitute, in fact, another mechanism chosen by tumor to promote local immune dysfunction and prevent effective response. We previously demonstrated that intratumoral nitration/nitrosylation of the chemokine CCL2 plays a crucial role in antitumor immunity, since RNS-modified CCL2 restrains T lymphocytes to the stroma at the border of neoplastic lesions, preventing their infiltration to the tumor core (64). According to this, preconditioning of tumor microenvironment with drugs impairing nitration induces massive T cell recruitment to tumor core and thus increases the efficacy of a T cell-based immunotherapy approach (64). Beyond this, alteration of l-arginine catabolism within tumors concomitantly causes local depletion of the amino acid (65). Deficiency in arginine availability affects protein synthesis in activated T cells, provoking a reduction in the expression of CD3ζ chain (66), activation markers, such as CD25, CD28, and CD62L, and has been shown to impair T cell proliferation and cytokine production (67).

Importantly, local depletion of another amino acid, tryptophan, alters T cell activity causing their anergy (68). Tryptophan deprivation within the tumor microenvironment is the result of the accelerated catabolism of the amino acid by the enzyme indoleamine 2,3-dioxygenase (IDO), which converts tryptophan to kynurenine and generates NAD. Although IDO enzymes are intracellular and not secreted, the metabolic effects of IDO are not locally confined to the IDO-expressing cells, but they rather involve neighboring cells that may sense and respond to the reduced availability of tryptophan and to secreted kynurenine metabolites (68). Importantly, although IDO activity may cause a significant decrease in tryptophan availability *in vitro*, a similar effect *in vivo*, where tryptophan concentration is 50–100 mM and a rapid diffusion from neighboring tissues is reasonably expected, has not been demonstrated so far (69). Therefore, even though the drop in tryptophan availability may not be sufficient to induce strong immunosuppressive effects, it may synergize with local accumulation of kynurenine and its derivatives to efficiently inhibit the proliferation and activation of immune cells (70). Based on studies examining serum levels of tryptophan and kynurenine, IDO appears to be chronically activated in patients with cancer, and IDO activation correlates with more extensive disease (71). IDO positivity has been indeed demonstrated in human tumors of different histology, such as colon and ovarian cancers, melanoma, and leukemia (72), and it is expressed by tumor cells (73), endothelial cells (74), and infiltrating immune cells, mainly APCs, DCs, and TAMs contributing to immune suppression (68, 75). Further, IDO-positive tumors have been demonstrated an impairment of T cell functions, since tryptophan shortage in the extracellular space induces T cell anergy and apoptosis (76). It has also been reported that antigen-specific cytotoxic T cells do not accumulate in the presence of IDO expressing tumor cells, but this tolerogenic process may be overcome, in mice, by the administration of the competitive IDO inhibitor 1MT, which is a tryptophan analog (77). Additionally, the administration of the small molecule, INCB024360, blocking IDO enzymatic activity in murine pancreatic ductal adenocarcinoma significantly inhibits tumor growth in a lymphocyte-dependent manner (78).

## Concluding Remarks

**Figure 1:**
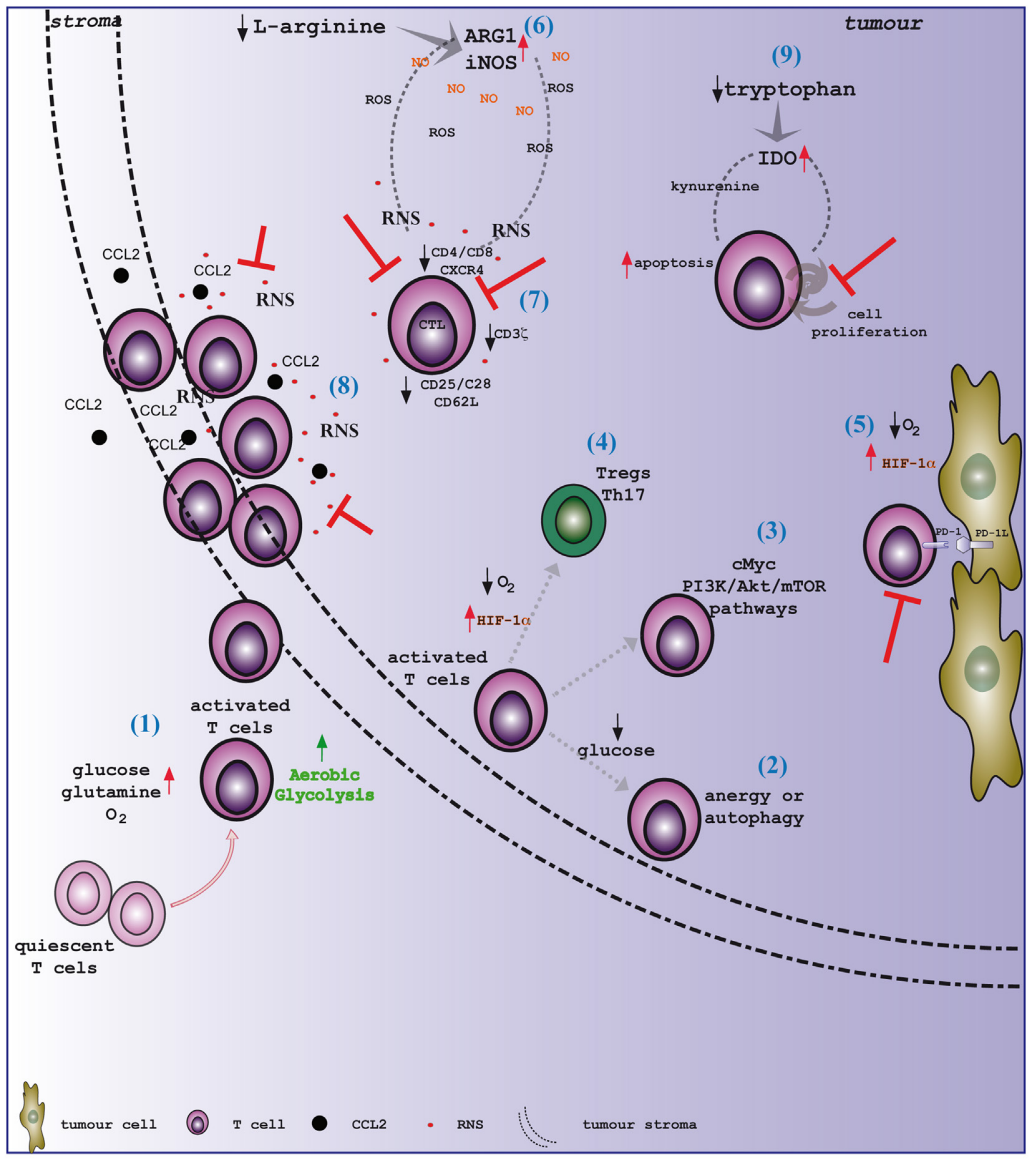
**Tumor microenvironment shapes T cell immunity**. (1) Activated T lymphocytes switch the metabolism toward aerobic glycolysis, increasing the uptake of glucose and glutamine from the outside and the consumption of oxygen. T cells, once reached the tumor site, need to face a hostile environment, which is characterized by hypoxia and nutrient deprivation. Distinct pathways regulate T cell metabolism within the tumor microenvironment. In particular, the drop of glucose level leads T cells to enter an (2) anergy state or to activate autophagy. Moreover, (3) c-Myc and the PI3K/Akt/mTOR pathways play a pivotal role in the energetic adaptation of T cells within transforming tissues. Tumor hypoxia sustains the activation of the transcription factor HIF-1α. HIF-1α represents a crucial metabolic checkpoint for (4) the differentiation of either T_reg_ or Th17 cells, and (5) it has been shown to upregulate the expression of PD-1 ligand on cancer cells. Remarkably, amino acid metabolism in tumors significantly impacts on T cell functions. Indeed, (6) the activation of both NOS and arginase enzymes in transforming tissues generates high levels of NO that rapidly react with ROS to generate RNS. (7) High and prolonged exposure to RNS promotes the downregulation of key proteins for T cell activation, such as the CD3ζ chain, CD25, CD28, and CD62L. Additionally, RNS induces the release of Ca^2+^ from intracellular stores, thus provoking a reduction in the expression of membrane receptors, such as CD4, CD8, and CXCR4. Moreover, (8) RNS-modified CCL2 restrains T lymphocytes to the stroma at the border of neoplastic lesions, preventing their infiltration to the tumor core. Tryptophan deprivation within the tumor microenvironment is mainly caused by the accelerated activity of the IDO enzyme. (9) The drop in tryptophan availability synergizes with the local accumulation of kynurenine and its derivatives to efficiently inhibit the proliferation and activation of T cells.

Metabolic reprograming of cancer and stromal cells in the tumor microenvironment is instrumental to meet the urgent need for energy supply to support tumor cell proliferation and progression, as well as immune evasion. The paucity of appropriate nutrients represents a limiting step for the effectiveness of antitumor T cell responses since T cells infiltrating malignant tissues need to face the tumor hostile environment to exert their functions (Figure 1). The deprivation of glucose and amino acids from the environment dramatically hinders T cell functions since it concomitantly induces the accumulation of toxic catabolic by-products. In this context, amino acid metabolism, and in particular l-arginine and tryptophan catabolism, represents a crucial process in tumor progression and immunity. The accumulation of RNS, dependent on the enhanced intratumoral metabolism of l-arginine within the tumor microenvironment, negatively affects T-cell-mediated immunity since it induces a dormant state in tumor-infiltrating lymphocytes, which have defects in signal transduction, migration, and effector killing capacity. Nonetheless, the administration of drugs impairing nitration positively influences T cell activation and cytotoxic activity and induces a massive T cell recruitment to tumor, thus increasing the efficacy of a T cell-based immunotherapy approach (64). Furthermore, pharmacological blockade of tryptophan catabolism results in the control of tumor growth in a lymphocyte-dependent manner. Collectively, interfering with amino acid metabolism within tumors represents a very promising option to develop novel combination therapy in order to rouse dormant T cells and prompt them to efficiently sustain tumor eradication.

## Author Contributions

BM, BC, and AV conceived and wrote the manuscript. BM and BC conceived and realized the figure.

## Conflict of Interest Statement

The authors declare that the research was conducted in the absence of any commercial or financial relationships that could be construed as a potential conflict of interest.
